# The incidence, operative difficulty and outcomes of staged versus index admission laparoscopic cholecystectomy and bile duct exploration for all comers: a review of 5750 patients

**DOI:** 10.1007/s00464-022-09272-0

**Published:** 2022-05-04

**Authors:** Silje Welsh, Ahmad H. M. Nassar, Mahmoud Sallam

**Affiliations:** Laparoscopic Biliary Service, University Hospital Monklands, Monkscourt Avenue, Airdrie, Lanarkshire ML6 0JS Scotland, UK

**Keywords:** Laparoscopic cholecystectomy, Bile duct exploration, Delayed cholecystectomy, Difficulty grading, Nassar scale, Index admission cholecystectomy

## Abstract

**Background:**

The timing of laparoscopic cholecystectomy (LC) for emergency biliary admissions remains inconsistent with national and international guidelines. The perception that LC is difficult in acute cholecystitis and the popularity of the two-session approach to pancreatitis and suspected choledocholithiasis result in delayed management.

**Methods:**

Analysis of prospectively maintained data in a unit adopting a policy of “intention to treat” during the index admission. The aim was to study the incidence of previous biliary admissions and compare the operative difficulty, complications and postoperative outcomes with patients who underwent index admission LC.

**Results:**

Of the 5750 LC performed, 20.8% had previous biliary episodes resulting in one admission in 93% and two or more in 7%. Most presented with biliary colic (39.6%) and acute cholecystitis (27.6%). A previous biliary history was associated with increased operative difficulty (*p* < 0.001), longer operating times (86.9 vs. 68.1 min, *p* < 0.001), more postoperative complications (7.8% vs. 5.4%, *p* = 0.002) and longer hospital stay (8.1 vs. 5.5 days, *p* < 0.001) and presentation to resolution intervals. However, conversion and mortality rates showed no significant differences.

**Conclusion:**

Index admission LC is superior to interval cholecystectomy and should be offered to all patients fit for general anaesthesia regardless of the presenting complaints. Subspecialisation should be encouraged as a major factor in optimising resource utilisation and postoperative outcomes of biliary emergencies.

Cholelithiasis and choledocholithiasis affect approximately 10–15% of the United Kingdom (UK) adult population [1, 2]. 50% of symptomatic patients will suffer recurring symptoms and 1–2% will develop serious complications [1]. Updated guidelines published in 2021 advocate proceeding with laparoscopic cholecystectomy within 7 days for all patients admitted with acute cholecystitis [1]. Similarly, laparoscopic cholecystectomy is recommended for all patients admitted with mild gallstone pancreatitis during the index admission or within 2 weeks of presentation [1]. Despite this, the longstanding practice of interval cholecystectomy with or without urgent biliary decompression in moderate pancreatitis with bile duct stones has been slow to change. The current common practice of preoperative endoscopic clearance of bile duct stones inevitably results in delay in performing LC in most cases. This study compares the outcomes in patients who received definitive treatment of stones in the gallbladder and the bile ducts, during the index admission, according to the protocol adopted by this biliary unit, to those requiring one or more admissions for various reasons, prior to being referred to the biliary unit. The staged treatment of acute cholecystitis and bile duct stones at other units or hospitals were major factors in delayed LC in most cases.

The primary aim was to study the incidence and causes of previous biliary admissions in a large series of patients undergoing LC and laparoscopic common bile duct exploration (LCBDE) admitted to a unit adopting index admission surgery. The secondary aim was to compare the operative difficulty, complications and postoperative outcomes of this group with patients who underwent LC and one-session LCBDE in a single admission.

## Materials and methods

A review and analysis was conducted of a single-surgeon database of prospectively collected parameters from 5750 laparoscopic cholecystectomies (LC) performed between February 1992 and October 2020. The database was interrogated for patient demographics, type and source of admission, clinical diagnosis, previous biliary history (including admissions, ERCP or surgical biliary procedures), diagnostic imaging, American Society of Anaesthesiologists (ASA) classification, operating time, the incidence of bile duct stones, conversion rate, perioperative complications and their management, 30-day re-operation, 30-day mortality, hospital stay, number of episodes and the total presentation to resolution interval. The intraoperative difficulty was scored according to the Nassar difficulty grading scale which describes 5 levels of difficulty according to the intraoperative findings of the gallbladder, cystic pedicle and presence of adhesions/fistulae [3–5]. Complications were classified according to the Clavien–Dindo classification system [6]. Where one patient experienced more than one complication, the highest Clavien–Dindo score was considered. All procedures were carried out by the senior surgeon (AHMN) or by his trainees in his presence under scrubbed on-table supervision.

Each discrete emergency and elective hospital admission secondary to symptomatic gallstone disease as a primary complaint was recorded as one episode. A biliary episode was defined as symptomatic gallstones leading to a hospital admission with biliary colic, cholecystitis, jaundice, pancreatitis and non-cardiac chest pain in the presence of gallstones. Patients were defined as having ‘previous biliary history’ when they had more than the one index admission episode during which they had a LC, excluding readmissions following surgery.

The referral pathways, operative techniques and postoperative management have been described in the previous studies [7, 8]. Emergency admissions with suspected biliary pathology undergo abdominal ultrasound and a plain X-ray of the chest. On confirmation of gallstone disease, the patient is referred to the dedicated biliary team with an intention to treat during the index admission. Those fit for general anaesthesia are offered LC with routine intraoperative cholangiography (IOC) and, if necessary, LCBDE. Preoperative computerised tomography scans of abdomen and pelvis (CTAP) and magnetic resonance cholangiopancreatography (MRCP) are reserved for patients with a high index of suspicion of hepatobiliary malignancy, as they are referred to specialist units upon confirmation. Patients with sepsis managed successfully within the first two or three days following admission were prepared for surgery subject to anaesthetic assessment. Some with severe or persistent sepsis precluding anaesthesia were managed conservatively.

Endoscopic retrograde cholangiopancreatography (ERCP) is reserved for patients with MRCP-confirmed CBD stones who are unsuitable for general anaesthesia. Some patients referred from medical or external firms may have already undergone cross-sectional imaging to assess patients with acute cholecystitis, pancreatitis or jaundice and to guide their management according to local protocols.

Subgroup analysis was performed according to the presence or absence of choledocholithiasis on the intraoperative cholangiogram.

Informed consent was obtained from all patients with emphasis on the specialisation of the unit with regard to the management of suspected bile duct stones. Data collection spanned 29 years and was carried out according to the requirements of the audit departments in the hospitals concerned. No ethical approval was required as the approved hospital protocols were in line with the guidelines and recommendations of national and international societies. The data were registered in the audit department and the study was limited to data analysis.

### Statistical analysis

Statistical analysis is performed by Pearson’s Chi-squared test for categorical variables. The normality of data is assessed by Shapiro–Wilk test. Thereafter, non-parametric continuous variables are analysed by Mann–Whitney U test and parametric by Student’s T test, as appropriate. A *p* value < 0.05 is considered statistically significant. Missing data were excluded from analysis. All analyses are performed using IBM SPSS 28.

## Results

A total of 5750 cholecystectomies were performed of which 1197 (20.8%) patients were recorded to have had previous biliary admissions. The mean age was 51.0 (± 16.0) years and there were 4248 (73.9%) female patients. However, males represented a significantly higher proportion of those who had a previous admission than those treated during the index admission (36.5% vs. 23.2% *p* < 0.001). 44.6% of all cholecystectomies in this study were performed during an emergency admission with no significant difference between those with index vs. previous admission. Table 1 summarises patient demographics, sources of referral, clinical presentations and preoperative MRCP and ERCP according to whether or not a previous biliary admission was recorded.

**Table 1:** Patient demographics and preoperative data

	No previous history IAC (*n* = 4553)	Previous history DLC (*n* = 1197)	P value
Sex			** < 0.001**
Females	3489 (76.8%)	759 (63.5%)	
Males	1056 (23.2%)	437 (36.5%)	
Unrecorded	Excluded (*n* = 1)	Excluded (*n* = 1)	
Mean age (SD), years	49.9 (15.8)	55.4 (16.1)	0.257
ASA classification			** < 0.001**
1	1677 (41.3%)	342 (31.9%)	
2	1854 (45.6%)	502 (46.8%)	
3	520 (12.8%)	223 (20.8%)	
4	14 (0.3%)	6 (0.6%)	
Unrecorded	Excluded (*n* = 488)	Excluded (*n* = 124)	
Procedure			0.096
Emergency	2055 (45.2%)	508 (42.5%)	
Elective	2495 (54.8%)	688 (57.5%)	
Unrecorded	*Excluded (n* = *2)*	*Excluded (n* = *2)*	
Source of referral			** < 0.001**
Other surgeons	1243 (27.3%)	529 (44.2%)	
Other hospital	783 (17.2%)	270 (22.5%)	
Physicians	119 (2.6%)	59 (4.9%)	
Self	2408 (52.9%)	339 (28.3%)	
Presenting complaint at time of IAC			
Chronic biliary colic	2570 (57.9%)	727 (60.7%)	**0.008**
Biliary colic	1409 (30.9%)	347 (29.0%)	0.191
Cholecystitis	417 (9.2%)	91 (7.6%)	0.091
Pancreatitis	366 (8.0%)	78 (6.5%)	0.079
Cholangitis	80 (1.8%)	53 (4.4%)	** < 0.001**
Jaundice	865 (19.0%)	191 (16.0%)	0.016
Chest pain	24 (0.5%)	8 (0.7%)	0.559
Preoperative MRCP	142 (3.1%)	174 (14.5%)	** < 0.001**
Preoperative ERCP^a^	41 (0.9%)	107(8.9%)	** < 0.001**

Statistically significant values are given in bold

Categorical variables analysed by Chi-squared test. Non-parametric continuous variables compared Mann–Whitney U and parametric by student’s T test as appropriate, as determined by Shapiro–Wilk test. A p value < 0.05 is considered statistically significant. Missing data were excluded from analysis

*NA* not applicable, *SD* standard deviation

^a^Including failed attempts and negative ERCPs

Biliary interventions during the previous admission were recorded in 11 of 1197 patients; one abandoned open cholecystectomy, three cholecystostomies (two open and one laparoscopic) and seven cholecystectomies with failed endoscopic clearance of the bile ducts necessitating surgical duct exploration. There were also 107 ERCPs (8.9%).

Of the 4553 patients who had index admission surgery, nine had undergone previous cholecystectomies many years earlier and had recurrent CBD stones with unsuccessful ERCP attempts requiring bile duct explorations. These were considered index admission procedures as there was no intention to treat in stages. Another 36 had preoperative ERCPs during the index episode.

Of the 1197 patients with previous biliary episodes, 60 patients did not undergo index admission cholecystectomy (IAC) according to their wishes (*n* = 6) or due to other care providers initially deeming patients unfit for surgical intervention (*n* = 54). The remaining 1137 patients had presented with symptomatic gallstones and received unsuccessful conservative management, 71.7% at other departments or hospitals. They were not referred to the biliary service during the initial admission (Table 1). Most had biliary colic (39.6%) or acute cholecystitis (27.6%) and 40% had jaundice or pancreatitis (Table 2). Acute pancreatitis (AP) accounted for 173/1197 (14.5%) of previous admissions. Of 444 AP, 7.7% of the whole series, 57 (12.8%) were recurrent episodes.

**Table 2:** Presentations of patients during previous biliary admissions

Previous biliary presentations (DLC)	*N* = 1197
Biliary colic	473 (39.6%)
Cholecystitis	329 (27.6%)
Jaundice	308 (25.8%)
Pancreatitis	173 (14.5%)
Chest pain	44 (3.7%)

The diagnoses of patients managed conservatively during previous hospital admissions

Some patients had more than one presenting symptom recorded

The mean number of total admissions per patient in those with previous biliary history was 2.08 (SD ± 0.330) (Table 3).

**Table 3:** Number of episodes in patients with a previous biliary admissions, including the index episode (*n* = 1197)

Total admissions	Number of patients
2	1115 (93.1%)
3	70 (5.8%)
4	8 (0.7%)
5	4 (0.3%)

The total number of episodes from first presentation with symptomatic gallstones to undergoing cholecystectomy

Trainees performed all or components of the LC or LCBDE, under on-table supervision, in 19.6% of those who had previous episodes and 23.9% who were operated upon during the index admission.

**Fig. 1:**
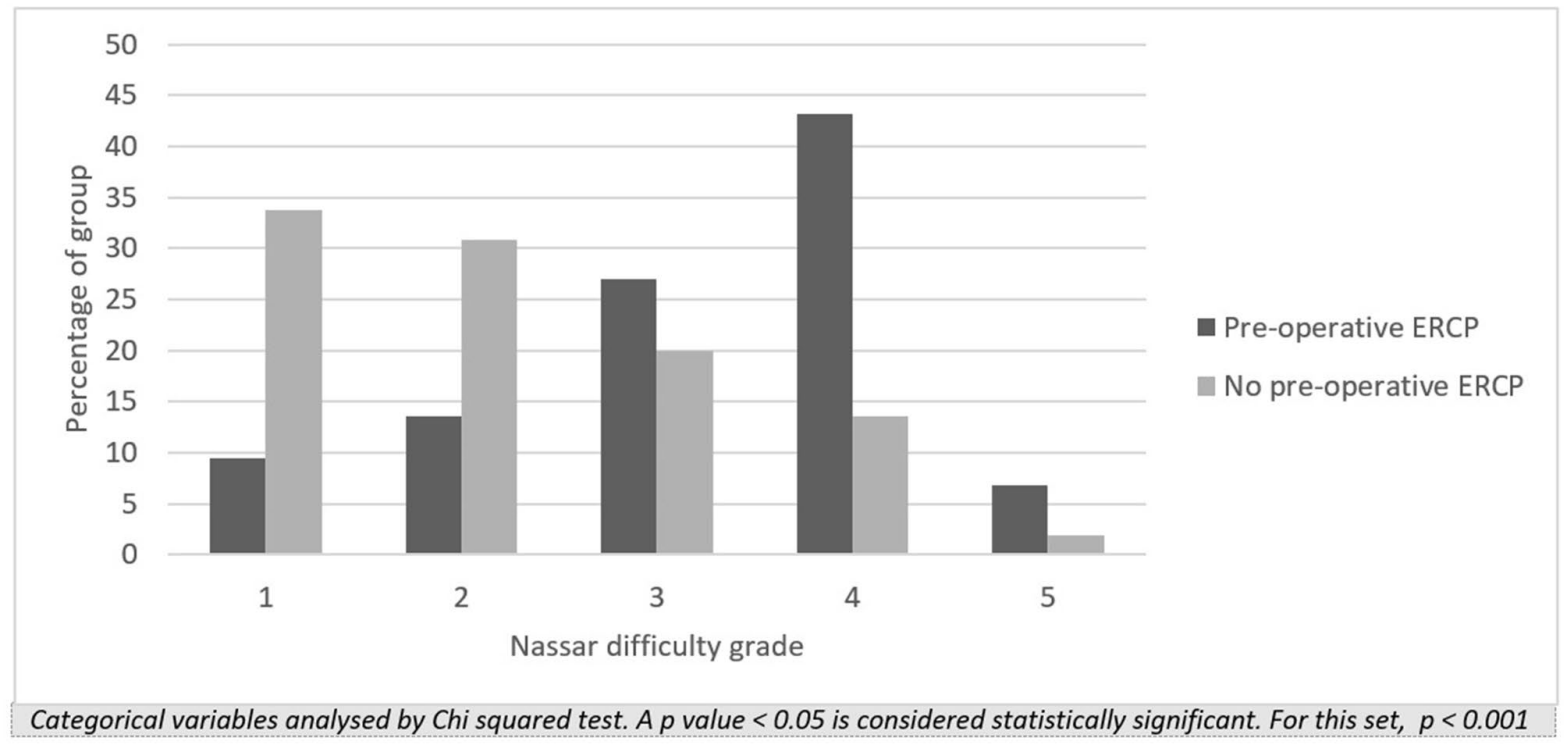
Operative difficulty grades in patients who underwent ERCP

Preoperative ERCP was associated with a significantly higher operative difficulty grade [3] and an increased risk of perioperative complications (Fig. 1). The incidence of complications in patients who had a preoperative ERCP was 16.9% compared to 5.4% for those with no preoperative ERCP (*p* < 0.001). Patients with previous biliary episodes also had a greater likelihood of increased operative difficulty (*p* < 0.001), required more intraoperative adhesiolysis, had a higher incidence of difficult cystic pedicles and required more fundus first dissection (*p* < 0.001). Having previous biliary episodes was significantly associated with a higher incidence of CBD stones (*p* < 0.001) and a longer mean operating time (*p* < 0.001). The rate of conversion to open (*p* = 0.138) was not different between the two groups (Table 4). Emergency cholecystectomy demonstrated a longer mean operating time of 87.0 (± 52.8) minutes compared to 59.9 (± 33.9) minutes for elective cases, *p* < 0.001. This difference remained significant whether or not the patients had bile duct stones and required LCBDE (mean operating times of 63.9 ± 37.7 and 79.1 ± 48.9 min with CBD stones and 69.3 ± 42.6 and 90.3 ± 57.2 min without CBD stones for patients without previous history compared to those with previous episodes, respectively, *p* < 0.001 for both).

**Table 4:** Operative findings in patients who underwent IAC and those who had DLC

	No previous episodes IAC (*n* = 4553)	Previous episodes DLC (*n* = 1197)	P value
Difficulty grading			** < 0.001**
I	1640 (36.0%)	262 (21.9%)	
II	1436 (31.5%)	311 (26.0%)	
III	857 (18.8%)	301 (25.1%)	
IV	556 (12.2%)	272 (22.7%)	
V	63 (1.4%)	51 (2.6%)	
Unrecorded	Excluded (*n* = 1)	0 (NA)	
Adhesiolysis			
Gallbladder	2763 (60.7%)	881 (73.6%)	** < 0.001**
Hepatic flexure	802 (17.6%)	353 (29.5%)	** < 0.001**
Duodenum	1971 (43.3%)	681 (56.9%)	** < 0.001**
Distant	456 (10.0%)	201 (16.8%)	** < 0.001**
Fundus first	104 (2.28%)	69 (5.76%)	** < 0.001**
Calot’s dissection			
Normal	3863 (80.9%)	856 (71.5%)	** < 0.001**
Easy	2902 (63.7%)	568 (47.5%)	** < 0.001**
Accessory cystic artery	1225 (26.9%)	416 (34.8%)	** < 0.001**
Wide cystic duct	530 (11.6%)	240 (20.1%)	** < 0.001**
Cystic duct stone	701 (15.4%)	252 (21.1%)	** < 0.001**
Mean operating time, minutes (SD)	68.1 (41.7)	86.9 (55.0)	** < 0.001**
Conversion to open	19 (0.4%)	9 (0.8%)	0.138
CBD stones	973 (21.4%)	363 (30.3%)	** < 0.001**
Management of CBD stones			0.072
Trans-cystic clearance	656 (67.4%)	224 (61.7%)	
CBDE	314 (32.3%)	139 (38.3%)	
Left in-situ	3 (0.3%)	0 (NA)	

Statistically significant values are given in bold

Categorical variables analysed by Chi-squared test. Non-parametric continuous variables compared Mann–Whitney U and parametric by students t test, as determined by Shapiro–Wilk test. A p value < 0.05 is considered statistically significant. Missing data were excluded from analysis

*NA* Not applicable, *SD* standard deviation

The post-operative complication rate in this study was 5.8% (337/5750) (excluding nausea and pain not resulting from specific complications or leading to longer hospital stays). Patients with previous biliary episodes were more likely to suffer morbidity (*p* = 0.002). However, this was not associated with a higher Clavien–Dindo classification, a greater risk of requiring re-operation, 30-day readmissions or a higher 30-day mortality rate. Fifty patients with Clavien–Dindo grade 3a complications include an incidence of post-cholecystectomy bile leakage and retained stones which has been presented in detail in previous studies [9, 10]. Within these 50 there were also four patients with postoperative collections drained percutaneously, two pneumothoraces (one due to intraoperative iatrogenic diaphragm injury and one from anaesthetic regional nerve blockade) managed by chest drain insertion and two patients with postoperative symptomatic pancreatitis.

Twenty one (0.4%) patients required re-operations: seven for bile leakage, six for T-tube complications, two for intra-abdominal abscesses and one for omental/port-site bleeding (all Clavien–Dindo grade 3b). The remaining two Clavien–Dindo grade 3b complications were CBD injuries which were referred to a national liver surgery unit for reconstruction. There were five Clavien–Dindo grade 4a complications. Two patients required re-intubation for respiratory failure secondary to chest infection, one patient underwent re-operation for perforated peptic ulcer disease and needed prolonged re-intubation and one had severe pancreatitis and confusion.

Four patients needed re-operations: two for mesenteric ischaemia, one iatrogenic colonic perforation and one for peritonitis secondary to a perforated undiagnosed colonic tumour and these four subsequently died. The remaining four deaths were due to sepsis from severe lower respiratory tract infections (*n* = 2), postoperative liver abscess and liver failure (*n* = 1) and one patient bled from an intrahepatic arterial aneurysm following attempted embolisation at another hospital (Clavien–Dindo grade 5).

Previous biliary admissions understandably resulted in more admission episodes per patient. Both the mean total hospital stay and the mean presentation to resolution interval were, therefore, significantly higher (Table 5).

**Table 5:** Postoperative data in patients with and without previous biliary emergency admissions

	No previous episodes IAC (*n* = 4553)	Previous episodes DLC (*n* = 1197)	P value
Total complications	244 (5.4%)	93 (7.8%)	**0.002**
Clavien–Dindo classification			0.375
1	100 (41.0%)	29 (31.2%)	
2	90 (36.9%)	37 (39.8%)	
3a	34 (13.9%)	16 (17.2%)	
3b	13 (5.3%)	5 (5.4%)	
4a	2 (0.8%)	3 (3.2%)	
5	5 (2.0%)	3 (3.2%)	
30-day readmission rate	149 (3.3%)	44 (3.7%)	0.491
Re-operation	14 (0.3%)	7 (0.6%)	0.157
30-day mortality	5 (0.1%)	3 (0.3%)	0.429
Mean no. of episodes/patient* (SD)	1.07 (0.3) (4869/4553)	2.08 (0.4) (2444/1197)	**0.006**
Mean hospital stay, days (SD)	5.5 (6.9) (in 3420)	8.1 (10.4) (in 881)	** < 0.001**
Mean presentation to resolution, weeks (SD)	1.9 (4.5) (in 3386)	6.9 (11.8) (in 888)	** < 0.001**

Statistically significant values are given in bold

Categorical variables analysed by Chi-squared test. Non-parametric continuous variables compared Mann–Whitney U and parametric by students T test as appropriate, as determined by Shapiro–Wilk test. A p value < 0.05 is considered statistically significant. Missing data were excluded from analysis

*NA* not applicable, *SD* standard deviation

^a^Including any readmissions

The presence of CBD stones on IOC did not result in significant differences in the incidence of complications, 30-day readmission rates, re-operation rates or 30-day mortality rates (Table 6).

**Table 6:** Choledocholithiasis detected on intraoperative cholangiogram in index admission vs. delayed cholecystectomy

	No previous episodes IAC (*n* = 973)	Previous episodes DLC (*n* = 363)	*P* value
Mean operating time, minutes (SD)	63.92 (37.705)	79.14 (48.920)	** < 0.001**
Complications	38 (0.04%)	20 (5.5%)	0.201
30-day readmission rate	29 (3.0%)	12 (3.3%)	0.759
Re-operation	4 (0.4%)	1 (0.3%)	0.718
30-day mortality	1 (0.4%)	1 (0.7%)	0.700
Mean no. of episodes/patient^a^ (SD)	1.06 (0.309)	2.04 (0.428)	**0.006**
Mean hospital stay, days (SD)	5.43 (7.265)	7.39 (9.489)	**0.002**
Mean presentation to resolution, weeks (SD)	1.89 (4.150)	7.01 (10.447)	** < 0.001**

Statistically significant values are given in bold

Categorical variables analysed by Chi-squared test. Non-parametric continuous variables compared Mann–Whitney U and parametric by students T test as appropriate, as determined by Shapiro–Wilk test. A p value < 0.05 is considered statistically significant. Missing data were excluded from analysis

*NA* not applicable, *SD* standard deviation

^a^Including any readmissions

## Discussion

This is a large study examining the effects of previous biliary admissions on the short-term outcomes of cholecystectomy. It does not only address patients presenting with acute cholecystitis (AC) but all comers with any biliary emergency. Index admission cholecystectomy (IAC) has traditionally been considered more technically challenging in AC with concerns about greater morbidity and higher conversion rates [11]. However, several meta-analyses have concluded that IAC is safe, without increased morbidity, and can significantly reduce the length of hospital stay when performed within seven days of admission for acute cholecystitis. The total length of hospital stay is reduced by between 3.7 and 8.2 days when IAC is offered when compared to medically managing the index admission and offering interval cholecystectomy [11–17]. The total stay is evidently longer as a result of repeat admissions, regardless of the hospital stay during the episode when LC is eventually performed, even as a day case. Index admission LC for AP, as recommended by the guidelines, optimised the utilisation of preoperative MRCP compared to those with previous pancreatitis (7.4% vs. 11.8%) and reduced the rate of ERCP (1.6% vs.7.7%). 77% of patients admitted with acute pancreatitis resolved in one episode, reflecting the benefits of the unit’s protocol. However, 43.2% of the previous episodes of AP were under the care of other surgeons, physicians or other hospitals and were not referred to the biliary unit at the time.

IAC is also associated with an overall reduction in cost per patient and improved patient satisfaction levels, albeit at the cost of a longer operating time as reported by some studies [12, 15, 16]. An additional benefit to IAC is that it negates the 9.7–20% risk of re-presentation with recurrent symptoms when patients with acute cholecystitis are discharged with a plan for interval cholecystectomy [12, 14]. These findings are supported by two meta-analyses. Wang et al*.* analysed the safety of early laparoscopic cholecystectomy (ELC) vs. delayed laparoscopic cholecystectomy (DLC) for cholecystitis with intercurrent mild pancreatitis. Of 426 patients (328 ELC and 98 DLC), hospital stay was significantly shorter and preoperative biliary events were less in the ELC group [18]. Zhong et al. analysed ten randomised controlled trials including a total of 1646 patients noting a significantly reduced risk of gallstone-related events in ELC compared to DLC (RR 0.17; 95% CI 0.07–0.44; *P* = 0.0003) [19]. The risk of interval complications leading to admission before LC is performed appears to be common to all presentations of gallstone disease.

### Index admission versus delayed cholecystectomy

Most patients with gallstone complications in this study (44.5%) underwent a cholecystectomy during an emergency index admission, with 79% of all cholecystectomies performed in one admission. The rate of “delayed” cholecystectomies of 20.1%, with more than a quarter of the previous episodes having occurred under the care of other hospitals or physicians, compares favourably with 36.8% of delayed cholecystectomies reported by the prospective CholeS study which captured current practice in 170 hospitals in the UK and Ireland during a two-month period in 2014 [20]. LC during an emergency admission was performed in only 15.8% in the CholeS cohort compared to the 44.5% in this study. The design of this biliary service, with a high workload of biliary emergencies, demonstrates the feasibility of IAC and optimises the clinical and cost outcomes within the recommended time frame of national guidelines [1, 2]. The feasibility of this policy was limited only by clinical fitness and by theatre availability in a small number of cases. Offering routine IOC (90% vs. 12% in CholeS) with CBD exploration (23.3% of all patients vs. 2.9% in the CholeS study) abolishes the delays associated with obtaining pre-operative MRCP, which was recorded in 5.5% and/or ERCP in 2.6% (vs. 26.1% and 10.8% in CholeS, respectively) [20]. Importantly, most of the pre-operative MRCP/ERCP observed in this study were obtained prior to referral to the biliary service. This study shows that IAC has a lower incidence of perioperative ERCP when compared to delayed cholecystectomy (0.9% vs. 8.9%) and this is consistent with the results of the Zhong et al. meta-analysis (2433 patients in 17 studies addressing ERCP usage) [19]. The safety of this single-stage approach with a high-volume emergency workload has previously been demonstrated [10].

Most patients with previous episodes had originally been admitted with biliary colic. These patients may require hospitalisation, in part, due to greater levels of pain in the context of reactive inflammatory processes. Therefore, effort should be concentrated on performing IAC in all suitable patients admitted with symptomatic gallstone disease, not only those with infective complications, pancreatitis or bile duct stones.

### Operating time

The shorter hospital stay of IAC is felt by many to be at the expense of longer operating times, suggesting a more complex operation, as concluded in two meta-analyses of 2000 patients [15, 16]. However, this study, with a much larger sample size, confirmed that IAC in the emergency setting results in significantly shorter mean operating times when compared to patients with previous biliary admissions (18.8 min less, *p* < 0.001). Delayed LC results in a technically more challenging procedure as demonstrated by higher Nassar difficulty grades and more intraoperative adhesiolysis observed in the ‘previous biliary history group’. This accounts for the longer operating times. While Zhong et al. and Wang et al*.* [18, 19] did not observe a significant difference in operating times between early and late cholecystectomies, the difference in this cohort may be accounted for by a significantly higher incidence of bile duct stones in delayed vs. early procedures (30.3% vs. 21.4%).

### Perioperative complications

This study observed a 7.8% morbidity rate in patients with a previous biliary history compared to 5.4% in those without (p < 0.002). Importantly, this was not reflected in the complication severity as defined by Clavien–Dindo classification scores, mortality rates or re-operation rates. These findings are in harmony with the outcome of a 2021 meta-analysis by Borzellino et al. showing that LC within 72 h of symptoms significantly reduces postoperative complications when compared to delayed cholecystectomy [21]. Conversely, Menahem et al. concluded in a 2015 meta-analysis that while ELC and DLC are equivalent in terms of overall morbidity, major bile duct injury and mortality, ELC was significantly associated with postoperative bile leaks and a higher incidence of intraoperative insertion of drainage tubes [22].

### Hospital stay

Unsurprisingly, this study demonstrated that patients undergoing DLC had significantly longer total hospital stay for all episodes than those who were offered index admission surgery. ELC has consistently been reported to have significantly shorter total hospital stays when compared to DLC [18, 19, 22].

### Presentation to resolution interval

The mean number of weeks from presentation to surgical intervention and resolution of all biliary problems for patients undergoing delayed cholecystectomy is on average over 5 weeks longer than those undergoing IAC. This undoubtedly brings additional negative effects on patient experience and satisfaction levels. A 2015 meta-analysis suggested that ELC would improve the quality of life and reduce the treatment cost for patients with acute cholecystitis when compared to those offered DLC [23]. The readmission rates in this study were not significantly different between the two groups, a finding in line with the meta-analysis by Zhong et al*.,* where nine studies including 1726 patients addressed the incidence of readmissions [19].

### Cholecystocholedocholithiasis

This can be managed in one-session or a two-stage approach depending primarily on the availability of trained surgeons or endoscopists as well as appropriate facilities and equipment. Delivery of this service also relies on the ability to coordinate the aforementioned variables. This study adopted a laparoscopic single-stage approach to suspected bile duct stones with surgical CBD exploration at the time of cholecystectomy thus avoiding repeat admissions and multiple treatment sessions. The presence of CBD stones on IOC and subsequent CBD exploration was not associated with additional morbidity or mortality in this study. Similarly, the operating times remained prolonged for patients with previous biliary episodes even in the absence of CBD stones. This suggests that previous biliary episodes increase the operative difficulty independently from the need to explore the bile duct. The management protocols, the logistics required for the service and the results of 1318 consecutive bile duct explorations performed by this unit over 28 years have been reported [24]. The superiority of single- over two-stage technique was also demonstrated in a 2015 meta-analysis including 8 randomised control trials and 1130 patients, finding improved stone clearance, reduced hospital stay and overall reduced operative time without additional morbidity or mortality [23]. A greater risk of de novo CBD stone formation has been observed when adopting the two-stage technique [25]. The incidental detection of malignancy of the gallbladder, biliary tree or pancreas during IOC has been described in a previous study of 1318 patients undergoing bile duct explorations [24]. In total, this series reported 26/5750 (0.45%) who were diagnosed with malignancy on IOC and choledochoscopy; 21 presenting with painful jaundice associated with acute cholecystitis or bile duct stones and no risk factors for malignancy and five without jaundice (four with previous episodes of cholecystitis). Seven patients had preoperative cross-sectional imaging, one with ERCP, which all showed no evidence of malignancy. Of the remaining 14 patients undergoing postoperative cross-sectional imaging following intraoperative findings, seven had radiologically undetectable disease, six had advanced disease treated palliatively and only one was suitable for a “curative resection”. It would seem that, based on the little diagnostic yield of curable biliary malignancies, a policy of no cross-sectional imaging in patients with obstructive jaundice is justifiable in units adopting single session management of bile duct stones.

This study’s observation that preoperative ERCP increases the difficulty of LC further substantiates the superiority of single-stage over two-stage management.

### Cost efficiency of early versus delayed LC

The findings of recent studies suggest that ELC may reduce healthcare costs and improve quality of life compared with DLC in patients with acute cholecystitis [25, 26]. The current study did not include the methodology or cost analysis of delayed cholecystectomies as most previous episodes were at other units or hospitals. However, the cost-effectiveness of emergency cholecystectomy for acute benign gallbladder disease in all comers (similar to this study) has been analysed by Sutton et al. [26] Using data from the CholeS study, a prospective population-based cohort study of the outcomes of cholecystectomy in the UK and Ireland, they concluded that emergency cholecystectomy was less costly (£4570 vs. £4720; €5484 vs. €5664) and, when using quality-adjusted life years (QALYs) as a unit of effectiveness, more effective (0.8868 vs. 0.8662 QALYs) than delayed cholecystectomy.

In conclusion, index admission laparoscopic cholecystectomy is clinically superior to interval cholecystectomy. Patients with previous biliary admissions have higher operative difficulty grades, longer operating times and greater perioperative complication rates. The single-stage laparoscopic approach to bile duct stones, when the expertise and facilities are available, adds to the benefits of early cholecystectomy. The reduced utilisation of preoperative MRCP and ERCP avoids unnecessary delays and reduces the cost of treatment. It may be that subspecialisation and the provision of urgent cholecystectomy are important factors in optimising short- and long-term outcomes in all comers with acute benign biliary conditions.
